# Amputation through the Shoulder Joint

**Published:** 1862-05

**Authors:** Chas. Winne


ART. IV.—*Amputation through the Shoulder Joint, by* Chas. Winne, M.D.

An Irish Emigrant on his journey west, fell from the platform of a rail road car, and his right arm was crushed by the last wheel, causing a compound comminuted fraction of the os humeri about the insertion of the deltoid muscle, and lacerating the integuments and muscles as it passed obliquely over it, down the upper part of the fore-arm—the patient was admitted into the Hospital of the Sisters of Charity, at Buffalo, on the same day. The necessity of immediate amputation of the arm at the seat of injury and fracture was obvious and strongly urged upon him and his friends, but was prohibited by both parties.

Severe inflammation and sphacelus of the parts below the fracture ensued; but youth, and a good constitution, and the most assiduous attention, saved him from impending death during the subsequent two weeks; he then acquiesced in the necessity of removing the arm, and desirous, if possible, to preserve the rotundity of the shoulder, and whatever might be saved of the shaft of the humerus, the first incision was made with this view. Upon raising the flap of the deltoid, the bone was found mostly denuded of periosteum up to the surgical neck, and upon opening the capsule of the joint, the synovial membrane and cartilage of the head, also, were found diseased. The whole of it was then removed. He bore the operation without sinking much, although at this time very much enfeebled by long suffering and exhaustive suppuration. The wound healed, for the most part, by first intention, and he rapidly recovered.

